# Canadian contributions to research on neglected tropical diseases

**DOI:** 10.1371/journal.pntd.0009476

**Published:** 2021-07-01

**Authors:** Theresa W. Gyorkos, Hélène Carabin, Maneesh Phillip, Leshawn Benedict, Amy Davis, Janet Hatcher Roberts, Kishor M. Wasan, Momar Ndao, Alison Krentel

**Affiliations:** 1 WHO Collaborating Centre for Research and Training in Parasite Epidemiology and Control, Department of Epidemiology, Biostatistics and Occupational Health, McGill University, Montreal, Canada; 2 Faculté de Médecine Vétérinaire, Université de Montréal, St-Hyacinthe, Canada; 3 École de Santé Publique de l’Université de Montréal, Montréal, Canada; 4 Centre de Recherche en Santé Publique (CReSP), Montréal, Canada; 5 Groupe de Recherche en Épidémiologie des Zoonoses et Santé Publique (GREZOSP), St-Hyacinthe, Canada; 6 Effect Hope, Markham, Canada; 7 Bruyère Research Institute, Ottawa, Canada; 8 School of Epidemiology and Public Health, University of Ottawa, Ottawa, Canada; 9 WHO Collaborating Centre for Knowledge Translation, Technology Assessment for Health Equity, Bruyère Research Institute, Ottawa, Canada; 10 Department of Urologic Sciences, Faculty of Medicine, University of British Columbia, Vancouver, Canada; 11 Neglected Global Diseases Initiative, University of British Colombia, Vancouver, Canada; 12 National Reference Centre for Parasitology, Research Institute of the McGill University Health Centre, Montreal, Canada; University of Calgary, CANADA

## Abstract

**Background:**

The World Health Organization’s (WHO) Neglected Tropical Disease (NTD) Road Map for 2021–2030 was recently endorsed by all member states at the World Health Assembly in November 2020. Although only 3 of the 20 NTDs are endemic in Canada (i.e., echinococcosis, rabies, and scabies), the Canadian research community has contributed to advancing the knowledge base of all 20 NTDs. Previous research comprehensively detailed Canadian research on 11 NTDs between 1950 and 2010 using a network analysis approach. The specific objective of the present analysis was to update the publication record over the last decade (2010–2019) to include all 20 NTDs.

**Materials and methods:**

A bibliometric analysis was conducted in Scopus and Web of Science databases (for English or French articles published between January 1, 2010 and December 31, 2019) using appropriate search terms for each of the 20 NTDs and where at least 1 of the authors had a Canadian institution address. A 21st search was added to include publications including multiple NTDs or a discussion of NTDs in general. Following assessment of inclusion and exclusion criteria, 2 reviewers independently screened all abstracts, with discordant observations rereviewed to arrive at an agreement. Duplicates were removed.

**Results:**

A total of 1,790 publications were retrieved (1,738 with a disease–specific NTD focus and 52 with a general NTD focus, resulting in 1,659 unique publications), giving an average of over 160 articles per year. Over 80% were classified as full–length research articles. The top 3 journals in terms of frequency were *PLOS Neglected Tropical Diseases*, *PLOS ONE*, and the *American Journal of Tropical Medicine and Hygiene*. Authors’ institutions were from all Canadian provinces. While all 20 NTDs were addressed in these publications, the 5 most commonly studied were leishmaniasis, dengue fever and chikungunya, Chagas disease, soil–transmitted helminthiases, and rabies.

**Conclusions:**

Canadian researchers across the country have contributed to the evidence base of all 20 NTDs, publishing an average of over 160 publications per year between 2010 and 2019. As WHO NTD Road Map 2021–2030 rolls out globally, the Canadian research community, in collaboration with its partners and in solidarity with people living in vulnerable circumstances in endemic regions worldwide, is well positioned to meet future research challenges so that the goal of eliminating the disease burden attributable to NTDs can be achieved.

## Introduction

Although there are personal accounts dating from the 1880s, the first published record of a neglected tropical disease (NTD) in humans in Canada is from 1937 [1,2]. It was a case of alveolar echinococcosis, presumably acquired in Iceland. Alveolar echinococcosis remains extremely rare in Canada; however, confirmed local transmission among canid hosts suggests its potential emergence in the human population, which is a matter of ongoing concern [3,4]. Cystic echinococcosis is much more widespread, being actively transmitted among wild and domesticated canid definitive hosts and cervid intermediate hosts in all but the Atlantic provinces [5]. While human cases of cystic echinococcosis have been reported from across Canada, endemicity is highest in northern and indigenous communities [5,6].

The only other NTDs endemic in Canada are rabies and scabies. Rabies is a federally reportable disease in both humans and animals. Human cases have declined over the years as vaccination programs in animals ramped up and are now extremely rare, with the last reported case in 2012 and the last death occurring in Alberta in 2007 following exposure to a rabid bat. Despite the vaccination programs, rabies continues to be transmitted primarily among wildlife (e.g., racoons, skunks, foxes, and bats), and cases, although rare, have also been reported among pets (e.g., dogs and cats) [7] and is of particular concern in the northern part of the country such as in Nunavik, Québec [8]. The epidemiology of scabies is largely unknown in Canada because it is not a reportable disease. The published literature documents cases in chronic health care institutions and indigenous communities; however, its importance as a current public health problem is unknown [9,10].

Researchers in Canada have not limited their investigations to these 3 NTDs. Indeed, Phillips and colleagues document contributions for 13 NTDs between 1950 and 2010 [11]. (These 13 NTDs would now be classified as 11 NTDs as roundworm, hookworm, and whipworm infections are considered together as 1 NTD [12].) There might be several reasons for a familiarity with all NTDs within the Canadian research community. They range from historical, and progressively more recent, collaborations with researchers outside of Canada, to increased travel and study abroad, to being mindful of the concept of the “global village,” a term coined by the famous Canadian writer, Marshall McLuhan in 1962 [13]. As a new decade in NTD research begins with the unanimous support of all World Health Assembly member states for the NTD Road Map 2021 to 2030 [12]—including Canada—the objective of this review is to appreciate the magnitude and scope of contributions made by the Canadian research community to NTDs over the last decade.

## Materials and methods

A total of 20 independent searches, 1 for each NTD, of Scopus and Web of Science were performed to identify peer-reviewed publications authored by at least 1 Canadian researcher (defined as specifying an institution in Canada as her/his affiliation in the publication) in the years 2010 to 2019, inclusive. The NTDs were Buruli ulcer, Chagas disease, dengue and chikungunya, dracunculiasis, echinococcosis, foodborne trematodiases, human African trypanosomiasis, leishmaniasis, leprosy, lymphatic filariasis, mycetoma and chromoblastomycosis and other deep mycoses, onchocerciasis, rabies, scabies and other ectoparasites, schistosomiasis, snakebite envenoming, soil-transmitted helminthiases, taeniasis/cysticercosis, trachoma, and yaws. The search terms included the NTD name, alternative NTD names, the associated pathogen, and specific symptoms of the NTD (when appropriate). Records retrieved from the 2 databases for each NTD were merged, and any duplicates were removed. Titles and abstracts were screened using the inclusion and exclusion criteria with the process managed using Covidence (covidence.org) (Table 1).

**Table 1:** Inclusion and exclusion criteria applied to each search in Scopus and Web of Science.

Inclusion criteria	Exclusion criteria
At least 1 author’s affiliation is a Canadian institution	
Publication date between January 1, 2010 and December 31, 2019	Nonspecific listing of the NTD within Global Burden of Disease or GeoSentinel or other large databases
Clear focus or subfocus on the NTD	Paper was retracted
Lab-based research on animal models, if link to the human NTD, is clear	Conference abstracts, proceedings, book chapters, and books
Language is English or French	
Full-text version is available	

NTD, neglected tropical disease.

Two independent reviewers reviewed the title and abstracts of each of the 20 searches. To ensure consistency, one of the reviewers was a reviewer for all 20 searches, while the second reviewer was selected based primarily on NTD subject expertise. In the event of any conflicts, the screeners discussed the findings and reviewed the full-text publication. Data were extracted into an Excel datasheet. During the 20 searches, it became clear that a 21st search was required so that publications having a more general overview of NTDs could be included. Descriptive statistics were computed using Microsoft Excel. The search strategies for all searches are shown in S1 Text.

## Results

**Fig 1:**
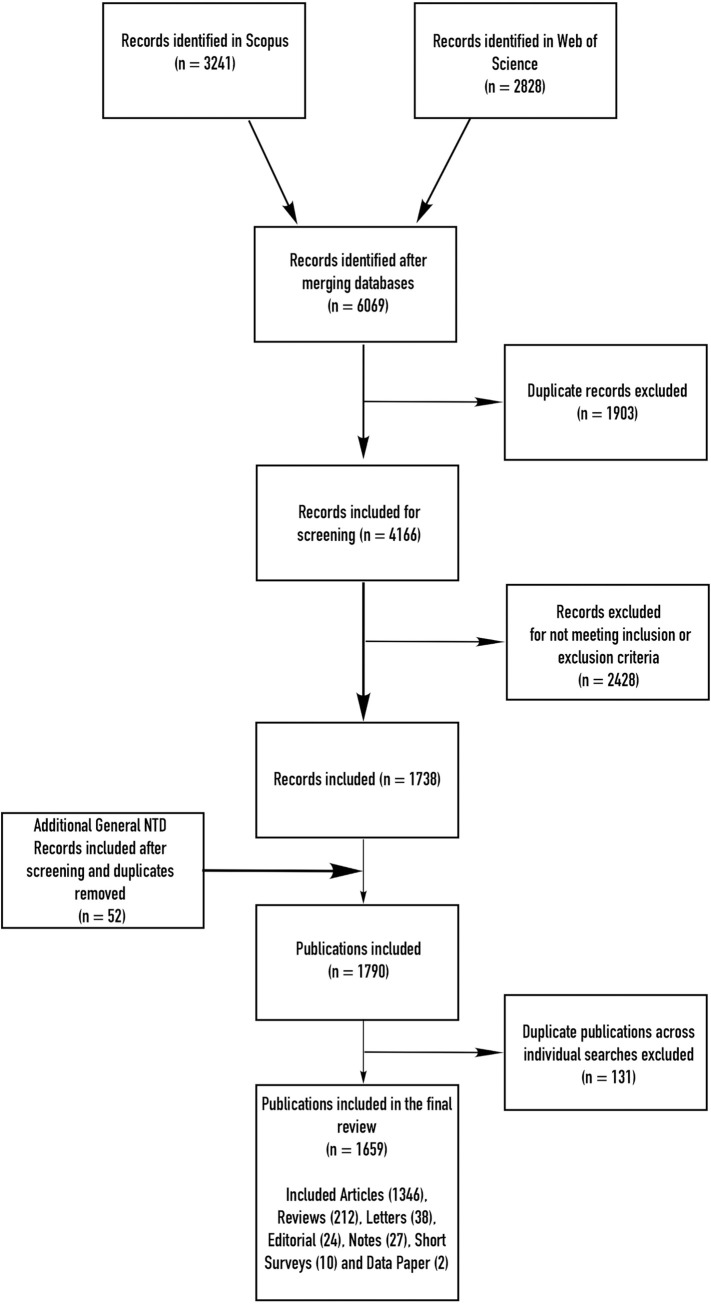
Combined results for 21 searches (the 20 NTDs independently, plus a search where NTDs were discussed in a general overarching way). NTD, neglected tropical disease.

**Fig 2:**
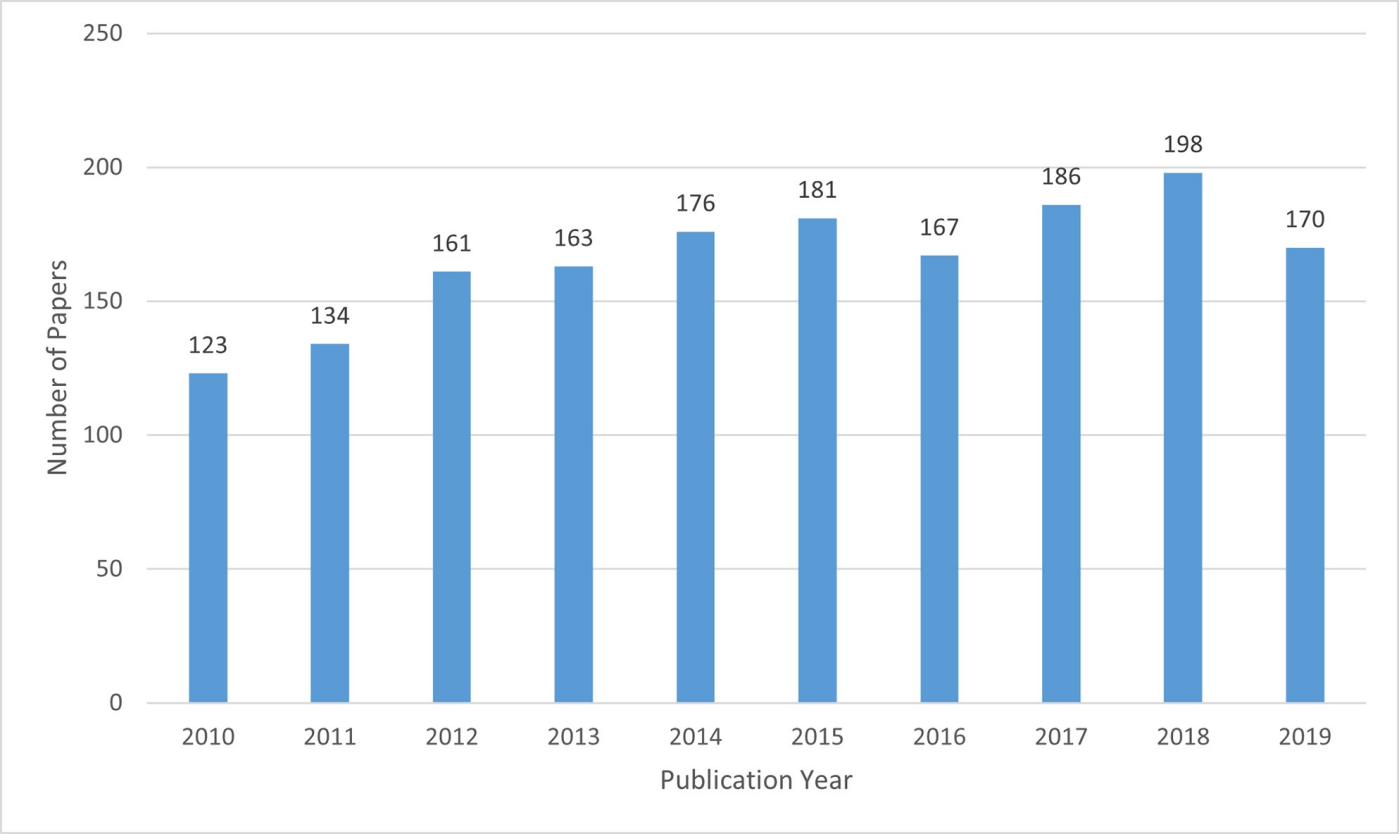
Canadian contributions to research on NTDs, 2010–2019 inclusive, by year. NTD, neglected tropical disease.

From the 20 independent searches that were conducted for each of the 20 NTDs and of a total number of 4,166 publications that were screened, a total of 1,738 publications were found after inclusion and exclusion criteria were met (Fig 1). An additional 52 publications were obtained from the 21st search where the content focused on NTDs in general rather than on any 1 disease. After duplicates were removed across individual searches (i.e., 131 publications had an in-depth focus on more than 1 NTD), the number of unique publications totalled 1,659, an average of over 165 publications per year (Fig 2). Fewer articles (less than 135) were published in 2010 and 2011 compared to the other years when more than 175 articles were published annually.

**Fig 3:**
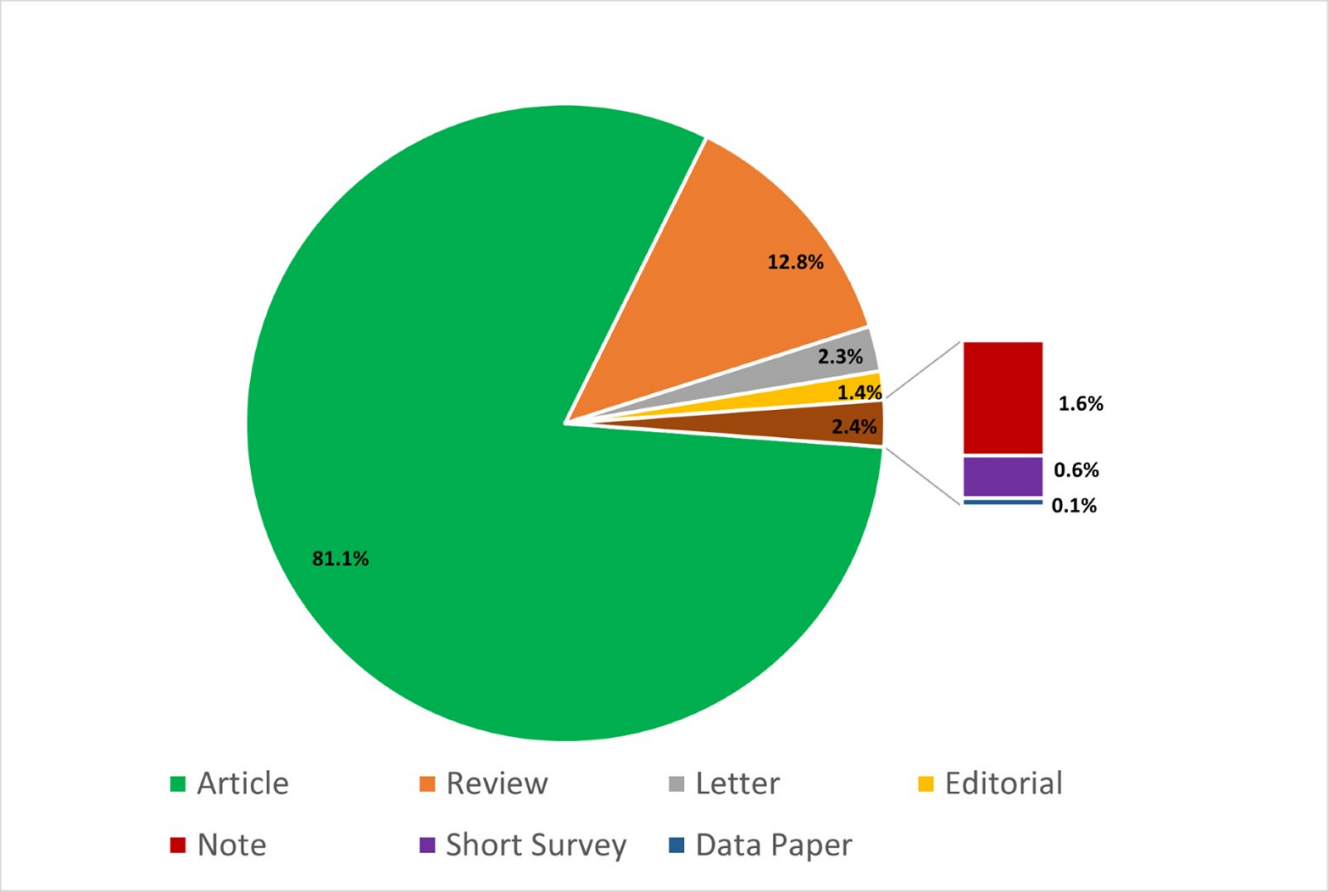
Canadian contribution to NTD publications by publication type (2010–2019). NTD, neglected tropical disease.

All Canadian provinces and the Northwest Territories were represented among the affiliations of at least 1 of the authors on the publications. The type of publication was categorized differently by each journal, but over 80% of the publications were classified as full-length research articles, 13% were reviews, and the balance included letters, editorials, notes, short surveys, and data papers (Fig 3).

**Fig 4:**
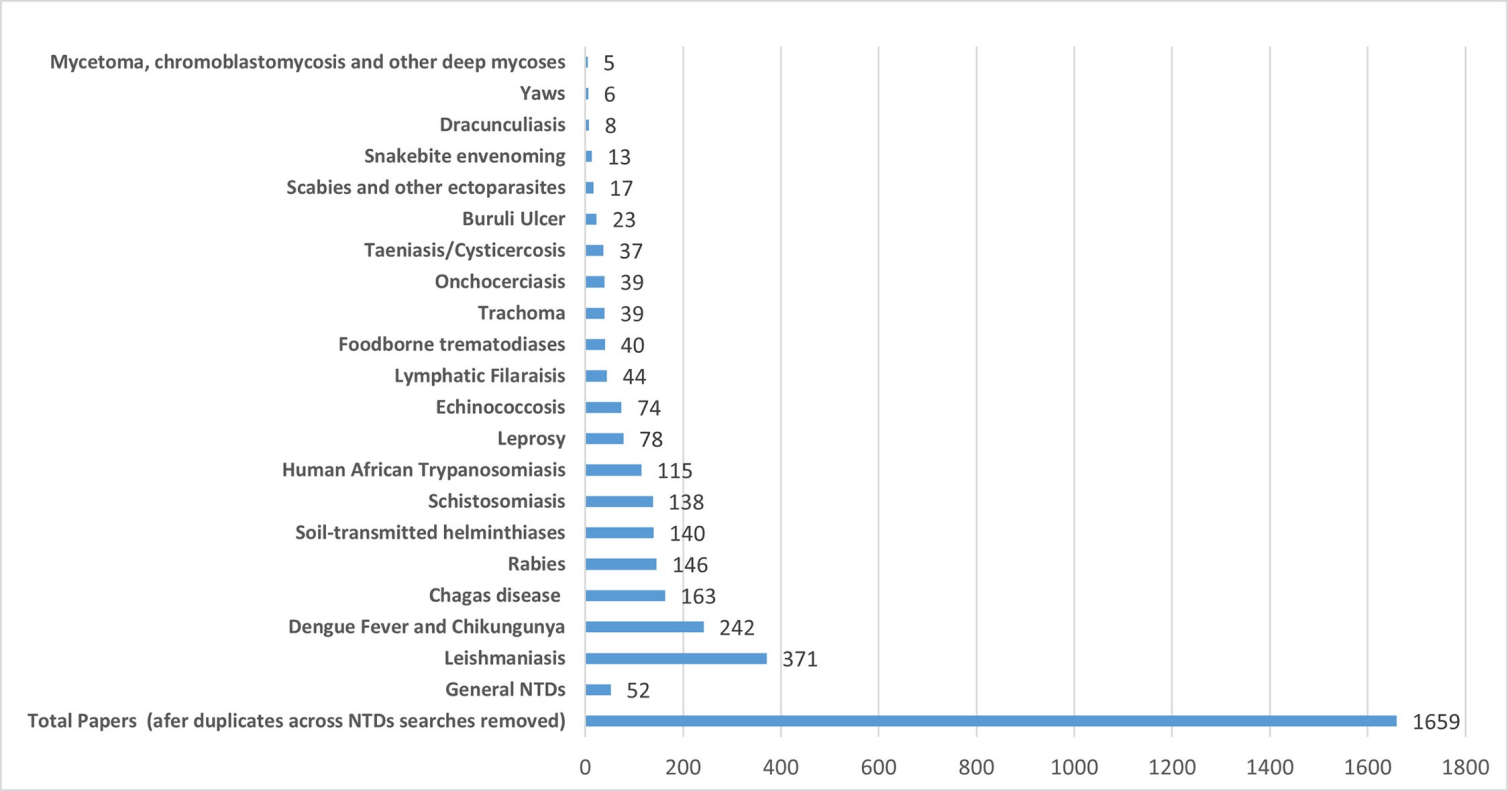
Canadian contributions to research on the 20 NTDs (2010–2019). NTD, neglected tropical disease.

In terms of cumulative published research over the study decade, leishmaniasis topped the list, followed by dengue and chikungunya and Chagas disease (Fig 4). Publications on mycetoma, chromoblastomycosis, and other deep mycoses, an NTD cluster that was only recently added to the World Health Organization’s (WHO) list of NTDs in 2017, garnered 5 publications.

This decade of Canadian research was published in a wide spectrum of scientific peer-reviewed journals (Table 2). Among the 549 journals in which this research was published, *PLOS Neglected Tropical Diseases* was the most frequently chosen (37%), followed by *PLOS ONE* (11%) and the *American Journal of Tropical Medicine and Hygiene* (10%).

**Table 2:** Journals where Canadian contributions to NTD research were published between January 1, 2010 and December 31, 2019.

Journal name	Number
**PLOS Neglected Tropical Diseases**	**196**
**PLOS ONE**	**62**
**American Journal of Tropical Medicine and Hygiene**	**53**
**PLOS Pathogens**	**37**
**Parasites & Vectors**	**26**
**Scientific Reports**	**24**
**Journal of Immunology**	**20**
**Molecular and Biochemical Parasitology**	**20**
**BMC Public Health**	**19**
**Vaccine**	**19**
**Other journals**	**1,183**
**TOTAL**	**1,659**

NTD, neglected tropical disease.

## Discussion

This bibliometric analysis provides some insight into the contribution Canadian researchers have made to advancing the knowledge base on all 20 NTDs over the last decade (2010 to 2019), extending the work of Phillips and colleagues, which summarized Canadian research on NTDs from 1950 to 2010 [11]. Importantly, it adds to this previous work by including 9 new NTDs, records from Web of Science, and records published in French. It illustrates the breadth and depth of this research across Canada. It also uncovers those diseases that have received the greatest attention (e.g., leishmaniasis). Reasons for this attention include historical and current links with institutions in endemic countries, interest in using an NTD as a model to delve into fundamental aspects of host–parasite interactions (especially once animal models became possible for some diseases (e.g., in leishmaniasis)), the zoonotic nature of some NTDs making infection in animals of interest to public health, particular interests of international students studying in Canada, and advocacy initiatives, especially with respect to the advancement of health equity and support for increased research funding [14,15].

There are many institutions in Canada that provide opportunities for research into NTDs. These include, among others, universities and colleges, specialized centers and institutes, nongovernmental organizations, and government departments. The recently created Canadian Network for NTDs (https://cnntd.org/) provides NTD-focused opportunities for student internships and for exchanges among Canadian partners and researchers (e.g., at the annual Canadian Conference on Global Health). As educational offerings in global health continue to expand across Canada and as initiatives to meet the Sustainable Development Goals gain momentum, it is likely that research into NTDs will trend upwards as well [16,17].

The research captured in this review provides some insight into the partnerships and collaborations that Canadian researchers have with colleagues in NTD-endemic countries. Although it was not possible to document the exact number of countries where research was carried out, it is clear that the reach of Canadian researchers extends from field work in Northern Canada to Latin America, Africa, and Asia and includes collaborations with global health organizations including United Nations organizations such as WHO, the Pan American Health Organization, WHO Special Programme for Research and Training in Tropical Diseases (WHO-TDR), the Food and Agriculture Organization, and the OIE (the World Organization for Animal Health), their regional offices, as well as civil society organizations based both in Canada and around the globe.

This review has several limitations. First, it should be emphasized that the Canadian research community that has conducted research on NTDs is much larger than that represented in the number of publications counted in this review. Many publications have more than 1 author from the same or different institutions in Canada, and many Canadian researchers are based in institutions in other countries and so would not be counted here. A more in-depth appreciation of the Canadian coauthorship network has been provided by Phillips and colleagues [11,18]. Updating these networks was beyond the scope of our analysis. Second, it may be that some potentially eligible publications were removed during screening because of an unclear title or abstract; the full text of some publications could not be accessed; and some publications that did include NTDs did so only as part of a much longer disease listing, without any NTD being a substantial focus (e.g., publications on the burden of disease or publications on the GeoSentinel database). Third, because of the magnitude of the results from our 21 searches, it was necessary to have several reviewers, and they may not have applied inclusion and exclusion criteria in a consistent manner. This latter limitation was mitigated to some extent by always having the same person as one of the reviewers.

It should be appreciated that this review does not include other forms of publication in which Canadian researchers have contributed to advancing research on NTDs (e.g., book chapters or books [19,20]; theses [18,21]; guides and reports [22,23]; and countless abstracts in proceedings of scientific meetings). Therefore, the cumulative research contribution documented here is certainly an underestimate. It is also important to mention that it was not possible to enumerate all the sources of funding, or the amount of funding, which supported this research. It may be that much of this funding has been obtained from external sources as there have been noted efforts to promote more Canadian funding for NTD research [14]. Additionally, there remain challenges in accessing data on global health research funding (and specifically on NTD funding) and in identifying the most appropriate metrics for meaningful analyses [11,24–26].

It should be recognized that attention to NTDs, and to the ultimate goal of reducing the mortality and morbidity associated with these diseases, has an impact much greater than any benefit accruing from the study of just one disease [27]. There have been important discoveries in drug development, in diagnostics, in disease burden assessment, and in preventive interventions, to name only a few. In tackling research gaps of NTDs, the Canadian research community, together with their global partners, contributes to reducing poverty, improving socioeconomic and educational status, empowering girls and women, and strengthening actions to provide universal access to good health for all peoples, globally. These contributions work to support the remit of the current Canadian overseas development assistance goals as outlined in the Feminist International Assistance Policy (FIAP). Further recognition in Canada of these research efforts and the intersection of NTDs with the FIAP are warranted, especially as the global community works toward more intersectoral approaches to health.

## Conclusions

Over the past decade, Canadian researchers have contributed to advancing the knowledge base of all 20 NTDs. This first bibliometric analysis quantifies this contribution and establishes the Canadian research community as an active and engaged partner in global actions to reduce the mortality, morbidity, and incalculable human suffering caused by NTDs, worldwide.

**Key learning points**

- Canadian researchers conduct research on all 20 neglected tropical diseases (NTDs).
- The most published NTDs are leishmaniasis, dengue and chikungunya, and Chagas disease.
- There are 3 NTDs endemic in Canada: echinococcosis, rabies, and scabies.
- The Canadian Network for NTDs brings together Canadians and Canadian organizations interested in helping reduce the suffering caused by NTDs worldwide.

**Top five papers**

1. World Health Organization. Ending the neglect to attain the Sustainable Development Goals: A road map for neglected tropical diseases 2021–2030. Geneva: World Health Organization; 2020.
2. Phillips K, Kohler JC, Pennefather P, Thorsteindottir H, Wong J. Canada’s neglected tropical disease research network: Who’s in the core–Who’s on the periphery? PLoS Negl Trop Dis. 2013;7(12):e2568. doi: 10.1371/journal.pntd.0002568
3. Krentel A, Gyapong M, Ogundahunsi O, Amuyunzu–Nyamongo, McFarland DA. Ensuring no one is left behind: Urgent action required to address implementation challenges for NTD control and elimination. PLoS Negl Trop Dis. 2018;12(6):e0006426. doi: 10.1371/journal.pntd.0006426
4. Gyorkos TW, Montresor A, Belizario V, Biggs B–A, Bradley M, Brooker SJ, et al. The right to deworming: The case for girls and women of reproductive age. PLoS Negl Trop Dis. 2018;12(11):e0006740. doi: 10.1371/journal.pntd.0006740
5. Booth M. Climate change and the neglected tropical diseases. Adv Parasitol. 2018;100:39-126. doi: 10.1016/bs.apar.2018.02.001

## Supporting information

S1 Text Search strategies for all 21 literature searches in Scopus and Web of Science. (DOCX)

## References

[1] James E, Boyd W. Echinococcus alveolaris. Can Med Assoc J. 1937;36(4):354–6. 20320583 PMC1562016

[2] Webster GA, Cameron TW. The epidemiology of echinococcosis in Canada. Can Med Assoc J. 1967;96:600–7. 6066987 PMC1936057

[3] Massolo A, Licciolo S, Budke C, Klein C. *Echinococcus multilocularis* in North America: the great unknown. Parasite. 2014;21(73). doi: 10.1051/parasite/2014069 25531581 PMC4273702

[4] Massolo A, Klein C, Kowalewska–Grochowska K, Belga S, MacDonald C, Vaughan S, et al. European *Echinococcus multilocularis* identified in patients in Canada. N Engl J Med. 2019;381(4):384–5. doi: 10.1056/NEJMc1814975 31340100

[5] Deplazes P, Rinaldi L, Alvarez Rojas CA, Torgerson PR, Harandi MF, Romig T, et al. Global distribution of alveolar and cystic echinococcosis. Adv Parasitol. 2017;95:315–493. doi: 10.1016/bs.apar.2016.11.001 28131365

[6] Gyorkos TW, MacLean JD, Serhir B, Ward B. Chapter 7 Prevalence of parasites in Canada and Alaska: epidemiology past and present. In: Akuffo H, Linder E, Ljungström I, Wahlgren M, editors. Parasites of the Colder Climates. London: Taylor & Francis; 2003.

[7] Public Health Agency of Canada. Diseases and conditions: Rabies. [cited 2020 Dec 3]. Available from: https://www.canada.ca/en/public–health/services/diseases/rabies/surveillance.html#a1.

[8] Mediouni S, Brisson M, Ravel A. Epidemiology of human exposure to rabies in Nunavik: incidence, the role of dog bites and their context, and victim profiles. BMC Public Health. 2020;20:584. doi: 10.1186/s12889-020-08606-8 32349705 PMC7191815

[9] Banerji A. Scabies. Paediatr Child Health. 2015;20(7):395. doi: 10.1093/pch/20.7.395 26527041 PMC4614097

[10] Holness DL, DeKoven JG, Nethercott JR. Scabies in chronic health care institutions. Arch Dermatol. 1992;128:1257–60. 1519942

[11] Phillips K, Kohler JC, Pennefather P, Thorsteindottir H, Wong J. Canada’s neglected tropical disease research network: Who’s in the core–Who’s on the periphery? PLoS Negl Trop Dis. 2013;7(12):e2568. doi: 10.1371/journal.pntd.0002568 24340113 PMC3854962

[12] World Health Organization. Neglected Tropical Diseases. Draft road map for neglected tropical diseases 2021–2030. Report by the Director–General 73rd World Health Assembly Provisional agenda item 118. A73/8, 2020.

[13] McLuhan M. The Gutenberg Galaxy: The making of typographic man. Toronto: University of Toronto Press; 1962.

[14] Gabriel P, Goulding R, Morgan–Jonker C, Turvey S, Nickerson J. Fostering Canadian drug research and development for neglected tropical diseases. Open Med. 2010;4(2):e117. 21709722 PMC3116685

[15] Nixon SA, Lee K, Bhutta ZA, Blanchard J, Haddad S, Hoffman SJ, et al. Canada’s global health role: supporting equity and global citizenship as a middle power. Lancet. 2018;391:1736–48. doi: 10.1016/S0140-6736(18)30322-2 29483026 PMC7138077

[16] Canadian Council for International Co–operation (CCIC). Transforming our world: Canadian perspectives on the Sustainable Development Goals. Ottawa: CCIC; 2016.

[17] Canadian Federation of Medical Students (CFMS). [cited 2020 Dec 11]. Available from: https://www.cfms.org/what–we–do/global–health/global–health–education.

[18] Phillips K. Canada’s contribution to neglected tropical disease research: A co–authorship network analysis. PhD thesis. University of Toronto; 2012.

[19] Mansfield J, Olivier M. Evasion and Latency: Evasion by parasites. In: Ahmed R, Sacks D, Kaufman SHE, editors. Immunology of Infectious Diseases. 2nd ed. Washington, DC: ASM Press; 2011.

[20] Potvin J–E, Leprohon P, Gazanion E, Sharma M, Fernandez–Prada C, Ouellette M. Chapter 7. Cos–Seq: A high throughput gain–of–function screen for drug resistance studies for *Leishmania*. In: Clos J, editor. *Leishmania* Methods and Protocols. New York: Humana Press; 2019. 10.1007/978-1-4939-9210-2_7 30980302

[21] Blouin B. Interrelationships between soil–transmitted helminth infections, hemoglobin levels and child development: a longitudinal cohort study. PhD thesis. McGill University; 2018.

[22] World Health Organization. Helminth Control in school–age children. A guide for managers of control programmes. 2nd ed. Geneva: World Health Organization; 2011.

[23] World Health Organization. Reaching girls and women of reproductive age with deworming. Report of the WHO Advisory Group on deworming in girls and women of reproductive age. Geneva: World Health Organization. WHO/CDS/NTD/PCT/2018.01.

[24] Gyorkos TW. Attempt to assess Canada’s expertise in global health research falls short. Health Res Policy Syst. 2020;18:130. doi: 10.1186/s12961-020-00634-5 33138844 PMC7607698

[25] Nagi R, Rogers Van Katwyk S, Hoffman SJ. Limitations in a rapid environmental scan of global health research expertise point to the need for more open data. Health Res Policy Syst. 2020;18:129. doi: 10.1186/s12961-020-00635-4 33138829 PMC7607651

[26] Nagi R, Rogers Van Katwyk S, Hoffman SJ. Using a rapid environmental scan methodology to map country–level global health research expertise in Canada. Health Res Policy Syst. 2020;18(37). doi: 10.1186/s12961-020-0543-x 32272941 PMC7146898

[27] Hotez PJ, Aksoy S, Brindley PJ, Kamhawi S. World neglected tropical diseases day. PLoS Negl Trop Dis. 2020;14(1):e0007999. doi: 10.1371/journal.pntd.0007999 31995572 PMC6988912

