# Supplementary material for: Canadian contributions to research on neglected tropical diseases
Source: PLoS Negl Trop Dis. 2021 Jul 1;15(7):e0009476. doi: 10.1371/journal.pntd.0009476 (PMC8248598; doi:10.1371/journal.pntd.0009476)
Supplement: S1 Text — (DOCX) [file pntd.0009476.s001.docx]

1. **Echinococcosis**

**Date of Search: September 16th, 2020**

**Scopus**

TITLE-ABS-KEY ( ( echinococcosis ) OR ("Cystic echinococcosis" ) OR ( "Alveolar echinococcosis" ) OR ( "Echinococcus granulosus" ) OR ( "Echinococcus multilocularis" ) OR ( "polycystic echinococcosis" ) OR ( "Echinococcus vogeli" ) OR ( "Echinococcus oligarthrus" ) OR ( "Echinococcus canadensis" ) OR ( "Echinococcus granulosus sensu lato" ) OR ( hydatidosis ) OR ( "hydatid disease" ) OR ( "Alveolar hydatid cyst" ) ) AND ( AFFILCOUNTRY ( canada ) ) AND ( LIMIT-TO ( PUBYEAR , 2019 ) OR LIMIT-TO ( PUBYEAR , 2018 ) OR LIMIT-TO ( PUBYEAR , 2017 ) OR LIMIT-TO ( PUBYEAR , 2016 ) OR LIMIT-TO ( PUBYEAR , 2015 ) OR LIMIT-TO ( PUBYEAR , 2014 ) OR LIMIT-TO ( PUBYEAR , 2013 ) OR LIMIT-TO ( PUBYEAR , 2012 ) OR LIMIT-TO ( PUBYEAR , 2011 ) OR LIMIT-TO ( PUBYEAR , 2010 ) ) AND ( LIMIT-TO ( PUBSTAGE , "final" ) ) AND ( LIMIT-TO ( LANGUAGE , "English" ) OR LIMIT-TO ( LANGUAGE , "French" ) ) AND ( LIMIT-TO ( SRCTYPE , "j" ) )

**Web of Science**

TOPIC: ((“Echinococcosis” OR “Cystic echinococcosis” OR “Alveolar echinococcosis” OR “Echinococcus granulosus” OR “Echinococcus multilocularis” OR “polycystic echinococcosis” OR “Echinococcus vogeli” OR “Echinococcus oligarthrus” OR “Echinococcus canadensis” OR “Echinococcus granulosus sensu lato” OR “hydatidosis” OR “hydatid disease” OR “Alveolar hydatid cyst”))

Refined by: PUBLICATION YEARS: ( 2019 OR 2018 OR 2017 OR 2016 OR 2015 OR 2014 OR 2013 OR 2012 OR 2011 OR 2010 ) AND COUNTRIES/REGIONS: ( CANADA ) AND [excluding] DOCUMENT TYPES: ( BOOK CHAPTER OR MEETING ABSTRACT ) AND LANGUAGES: ( ENGLISH )

Timespan: All years. Indexes: SCI-EXPANDED, SSCI, A&HCI, CPCI-S, CPCI-SSH, BKCI-S, BKCI-SSH, ESCI, CCR-EXPANDED, IC.

1. **Lymphatic Filariasis**

**Date of Search: September 16th, 2020**

**Scopus**

TITLE-ABS-KEY ( ( "Lymphatic filariasis" OR "Wuchereria bancrofti" OR "Brugia malayi" OR "Brugia timori" OR "elephantiasis" OR "Elephantiasis, Filarial" OR "Filarial Elephantiasis" OR "Bancroftian Elephantiasis" OR "Elephantiases" OR "Filarial Elephantiases" OR "Filariasis, Lymphatic" OR "Filariases, Lymphatic" OR "Lymphatic Filariases" OR "Brugia malayi" ) ) AND ( AFFILCOUNTRY ( canada ) ) AND ( LIMIT-TO ( PUBYEAR , 2019 ) OR LIMIT-TO ( PUBYEAR , 2018 ) OR LIMIT-TO ( PUBYEAR , 2017 ) OR LIMIT-TO ( PUBYEAR , 2016 ) OR LIMIT-TO ( PUBYEAR , 2015 ) OR LIMIT-TO ( PUBYEAR , 2014 ) OR LIMIT-TO ( PUBYEAR , 2013 ) OR LIMIT-TO ( PUBYEAR , 2012 ) OR LIMIT-TO ( PUBYEAR , 2011 ) OR LIMIT-TO ( PUBYEAR , 2010 ) ) AND ( LIMIT-TO ( PUBSTAGE , "final" ) ) AND ( LIMIT-TO ( LANGUAGE , "English" ) OR LIMIT-TO ( LANGUAGE , "French" ) ) AND ( LIMIT-TO ( SRCTYPE , "j" ) )

**Web of Science**

TOPIC: ((“Lymphatic filariasis” OR “Wuchereria bancrofti” OR “Brugia malayi” OR “Brugia timori” OR “elephantiasis” OR “Elephantiasis, Filarial” OR “Filarial Elephantiasis” OR “Bancroftian Elephantiasis” OR “Elephantiases” OR “Filarial Elephantiases” OR “Filariasis, Lymphatic” OR "Filariases, Lymphatic" OR "Lymphatic Filariases" OR "Brugia malayi"))

Refined by: PUBLICATION YEARS: ( 2019 OR 2018 OR 2017 OR 2016 OR 2015 OR 2014 OR 2013 OR 2012 OR 2011 OR 2010 ) AND DOCUMENT TYPES: ( ARTICLE OR LETTER OR CORRECTION OR REVIEW OR EDITORIAL MATERIAL OR NEWS ITEM ) AND COUNTRIES/REGIONS: ( CANADA ) AND LANGUAGES: ( ENGLISH OR FRENCH )

Timespan: All years. Indexes: SCI-EXPANDED, SSCI, A&HCI, CPCI-S, CPCI-SSH, BKCI-S, BKCI-SSH, ESCI, CCR-EXPANDED, IC.

**3) Onchocerciasis**

**Date of Search: September 17th, 2020**

**Scopus**

TITLE-ABS-KEY ( ( "Onchocerciasis" OR "River blindness" OR "Robles disease" OR "Onchocerca volvulus" OR "Erisipela de laCosta" OR "Mal morando" OR "Sowda" OR "Ocular Onchocercias*" OR "Ocular Onchocerciasis" ) ) AND ( AFFILCOUNTRY ( canada ) ) AND ( LIMIT-TO ( PUBYEAR , 2019 ) OR LIMIT-TO ( PUBYEAR , 2018 ) OR LIMIT-TO ( PUBYEAR , 2017 ) OR LIMIT-TO ( PUBYEAR , 2016 ) OR LIMIT-TO ( PUBYEAR , 2015 ) OR LIMIT-TO ( PUBYEAR , 2014 ) OR LIMIT-TO ( PUBYEAR , 2013 ) OR LIMIT-TO ( PUBYEAR , 2012 ) OR LIMIT-TO ( PUBYEAR , 2011 ) OR LIMIT-TO ( PUBYEAR , 2010 ) ) AND ( LIMIT-TO ( PUBSTAGE , "final" ) ) AND ( LIMIT-TO ( LANGUAGE , "English" ) ) AND ( LIMIT-TO ( SRCTYPE , "j" ) )

**Web of Science**

TOPIC: ((“Onchocerciasis” OR “River blindness” OR “Robles disease” OR “Onchocerca volvulus” OR “Erisipela de la Costa” OR "Mal morando" OR "Sowda" OR "Ocular Onchocercias*” OR "Ocular Onchocerciasis”))

Refined by: PUBLICATION YEARS: ( 2019 OR 2018 OR 2017 OR 2016 OR 2015 OR 2014 OR 2013 OR 2012 OR 2011 OR 2010 ) AND COUNTRIES/REGIONS: ( CANADA ) AND LANGUAGES: ( ENGLISH ) AND [excluding] DOCUMENT TYPES: ( BOOK CHAPTER OR PROCEEDINGS PAPER OR MEETING ABSTRACT )

Timespan: All years. Indexes: SCI-EXPANDED, SSCI, A&HCI, CPCI-S, CPCI-SSH, BKCI-S, BKCI-SSH, ESCI, CCR-EXPANDED, IC.

**4) Trachoma**

**Date of Search: September 17th, 2020**

**Scopus**

TITLE-ABS-KEY ( ( "Trachoma" OR "Chlamydia trachomatis" OR "Granular conjunctivitis" OR "Blinding Trachmoa" OR "trichiasis" OR "Egyptian Ophthalmia" OR "Ophthalmia, Egyptian" OR "Conjunctivitis - granular" ) ) AND ( AFFILCOUNTRY ( canada ) ) AND ( LIMIT-TO ( PUBYEAR , 2019 ) OR LIMIT-TO ( PUBYEAR , 2018 ) OR LIMIT-TO ( PUBYEAR , 2017 ) OR LIMIT-TO ( PUBYEAR , 2016 ) OR LIMIT-TO ( PUBYEAR , 2015 ) OR LIMIT-TO ( PUBYEAR , 2014 ) OR LIMIT-TO ( PUBYEAR , 2013 ) OR LIMIT-TO ( PUBYEAR , 2012 ) OR LIMIT-TO ( PUBYEAR , 2011 ) OR LIMIT-TO ( PUBYEAR , 2010 ) ) AND ( LIMIT-TO ( PUBSTAGE , "final" ) ) AND ( LIMIT-TO ( DOCTYPE , "ar" ) OR LIMIT-TO ( DOCTYPE , "re" ) OR LIMIT-TO ( DOCTYPE , "le" ) OR LIMIT-TO ( DOCTYPE , "ch" ) OR LIMIT-TO ( DOCTYPE , "ed" ) OR LIMIT-TO ( DOCTYPE , "no" ) OR LIMIT-TO ( DOCTYPE , "sh" ) OR LIMIT-TO ( DOCTYPE , "Undefined" ) ) AND ( LIMIT-TO ( LANGUAGE , "English" ) OR LIMIT-TO ( LANGUAGE , "French" ) ) AND ( LIMIT-TO ( SRCTYPE , "j" ) )

**Web of Science**

TOPIC: (( “Trachoma” OR “Chlamydia trachomatis” OR “Granular conjunctivitis” OR "Blinding Trachmoa" OR "trichiasis" OR "Egyptian Ophthalmia" OR "Ophthalmia, Egyptian" OR "Conjunctivitis - granular"))

Refined by: PUBLICATION YEARS: ( 2019 OR 2018 OR 2017 OR 2016 OR 2015 OR 2014 OR 2013 OR 2012 OR 2011 OR 2010 ) AND COUNTRIES/REGIONS: ( CANADA ) AND [excluding] DOCUMENT TYPES: ( MEETING ABSTRACT OR BOOK CHAPTER OR PROCEEDINGS PAPER ) AND LANGUAGES: ( ENGLISH OR FRENCH )

Timespan: All years. Indexes: SCI-EXPANDED, SSCI, A&HCI, CPCI-S, CPCI-SSH, BKCI-S, BKCI-SSH, ESCI, CCR-EXPANDED, IC.

**5) Schistosomiasis**

**Date of Search: September 17th, 2020**

**Scopus**

TITLE-ABS-KEY ( ( ( "Schistosomiasis" OR "bilharzia*" OR "Schistosoma mansoni" OR "Schistosoma haematobium" OR "Schistosoma japonicum" OR "Katayama fever" OR "Schistosoma haematobia" OR "S. Haematobium" OR "Schistosoma mansoni" OR "S. Mansoni" OR "S. Japoni*" OR "S indicum" OR "S. guineensis" OR "S. intercalatum" OR "Schistosoma mekongi" OR "S. Mekongi" OR "Neuroschistosomiasis" OR "Bilharziasis" OR "Bilharziases" OR "Schistosomiases" OR "Schistosomiasis, Urinary" OR "Urinary Schistosomiasis" OR "Schistosomiasis, Intestinal" OR "Intestinal Schistosomiases" OR "Intestinal Schistosomiasis" OR "Schistosomiases, Intestinal" OR "Neuroschistosomiases" OR "Central Nervous SystemSchistosomiasis" OR "Schistosomiasis, Central Nervous System" OR "SchistosomalMyelopathy" OR "Myelopathies, Schistosomal" OR "Myelopathy, Schistosomal" OR "Schistosomal Myelopathies" OR "Schistosomal Myeloradiculopathy" OR "Myeloradiculopathies, Schistosomal" OR "Myeloradiculopathy, Schistosomal" OR "Schistosomal Myeloradiculopathies" OR "Schistosomal Myelitis" OR "Myelitis, Schistosomal" OR "Schistosoma" OR "schistosomes" ) ) ) AND ( AFFILCOUNTRY ( canada ) ) AND ( LIMIT-TO ( PUBYEAR , 2019 ) OR LIMIT-TO ( PUBYEAR , 2018 ) OR LIMIT-TO ( PUBYEAR , 2017 ) OR LIMIT-TO ( PUBYEAR , 2016 ) OR LIMIT-TO ( PUBYEAR , 2015 ) OR LIMIT-TO ( PUBYEAR , 2014 ) OR LIMIT-TO ( PUBYEAR , 2013 ) OR LIMIT-TO ( PUBYEAR , 2012 ) OR LIMIT-TO ( PUBYEAR , 2011 ) OR LIMIT-TO ( PUBYEAR , 2010 ) ) AND ( EXCLUDE ( DOCTYPE , "ch" ) OR EXCLUDE ( DOCTYPE , "cp" ) OR EXCLUDE ( DOCTYPE , "bk" ) ) AND ( LIMIT-TO ( PUBSTAGE , "final" ) ) AND ( LIMIT-TO ( LANGUAGE , "English" ) OR LIMIT-TO ( LANGUAGE , "French" ) ) AND ( LIMIT-TO ( SRCTYPE , "j" ) )

**Web of Science**

TOPIC: ((“Schistosomiasis” OR “bilharzia*” OR “Schistosoma mansoni” OR “Schistosoma haematobium” OR “Schistosoma japonicum” OR "Katayama fever" OR "Schistosoma haematobia" OR "S. Haematobium" OR “Schistosoma mansoni" OR "S. Mansoni" OR “S. Japoni*" OR "S indicum" OR "S. guineensis" OR "S. intercalatum" OR "Schistosoma mekongi" OR "S. Mekongi" OR "Neuroschistosomiasis" OR "Bilharziasis" OR "Bilharziases" OR "Schistosomiases" OR "Schistosomiasis, Urinary" OR "Urinary Schistosomiasis" OR "Schistosomiasis, Intestinal" OR "Intestinal Schistosomiases" OR "Intestinal Schistosomiasis" OR "Schistosomiases, Intestinal" OR "Neuroschistosomiases" OR "Central Nervous System Schistosomiasis" OR "Schistosomiasis, Central Nervous System" OR "Schistosomal Myelopathy" OR "Myelopathies, Schistosomal" OR "Myelopathy, Schistosomal" OR "Schistosomal Myelopathies" OR "Schistosomal Myeloradiculopathy" OR "Myeloradiculopathies, Schistosomal" OR "Myeloradiculopathy, Schistosomal" OR "Schistosomal Myeloradiculopathies" OR "Schistosomal Myelitis" OR "Myelitis, Schistosomal" OR "Schistosoma" OR "schistosomes"))

Refined by: PUBLICATION YEARS: ( 2019 OR 2018 OR 2017 OR 2016 OR 2015 OR 2014 OR 2013 OR 2012 OR 2011 OR 2010 ) AND [excluding] DOCUMENT TYPES: ( PROCEEDINGS PAPER OR MEETING ABSTRACT OR BOOK CHAPTER OR BOOK OR BOOK REVIEW ) AND COUNTRIES/REGIONS: ( CANADA ) AND LANGUAGES: ( ENGLISH )

Timespan: All years. Indexes: SCI-EXPANDED, SSCI, A&HCI, CPCI-S, CPCI-SSH, BKCI-S, BKCI-SSH, ESCI, CCR-EXPANDED, IC.

**6) Buruli Ulcer**

**Date of Search: September 17th, 2020**

**Scopus**

TITLE-ABS-KEY ( ( "Buruli Ulcer*" OR "Mycobacterium ulcerans" OR "Infection, Mycobacterium ulcerans" OR "Ulcer, Buruli" ) ) AND ( AFFILCOUNTRY ( canada ) ) AND ( LIMIT-TO ( PUBYEAR , 2019 ) OR LIMIT-TO ( PUBYEAR , 2018 ) OR LIMIT-TO ( PUBYEAR , 2017 ) OR LIMIT-TO ( PUBYEAR , 2016 ) OR LIMIT-TO ( PUBYEAR , 2015 ) OR LIMIT-TO ( PUBYEAR , 2014 ) OR LIMIT-TO ( PUBYEAR , 2013 ) OR LIMIT-TO ( PUBYEAR , 2012 ) OR LIMIT-TO ( PUBYEAR , 2011 ) OR LIMIT-TO ( PUBYEAR , 2010 ) ) AND ( LIMIT-TO ( PUBSTAGE , "final" ) ) AND ( LIMIT-TO ( LANGUAGE , "English" ) OR LIMIT-TO ( LANGUAGE , "French" ) ) AND ( LIMIT-TO ( SRCTYPE , "j" ) )

**Web of Science**

TOPIC: (“Buruli Ulcer*” OR “Mycobacterium ulcerans” OR "Infection, Mycobacterium ulcerans" OR "Ulcer, Buruli")

Refined by: PUBLICATION YEARS: ( 2019 OR 2018 OR 2017 OR 2016 OR 2015 OR 2014 OR 2013 OR 2012 OR 2011 OR 2010 ) AND [excluding] DOCUMENT TYPES: ( BOOK CHAPTER OR PROCEEDINGS PAPER OR MEETING ABSTRACT ) AND COUNTRIES/REGIONS: ( CANADA ) AND LANGUAGES: ( ENGLISH OR FRENCH )

Timespan: All years. Indexes: SCI-EXPANDED, SSCI, A&HCI, CPCI-S, CPCI-SSH, BKCI-S, BKCI-SSH, ESCI, CCR-EXPANDED, IC.

**7) Chagas disease (American trypanosomiasis)**

**Date of Search: September 17th, 2020**

**Scopus**

TITLE-ABS-KEY ( ( "Chagas disease" OR "American trypanosomiasis" OR "Trypanosoma cruzi" OR "Triatominae" OR "kissing bugs" OR "T. cruzi Infection" OR "South American Trypanosomiasis" OR "Disease, Chagas" OR "Trypanosomiasis, American" OR "Trypanosomiasis, South American" OR "Chagas' Disease" OR "Trypanosoma cruzi" ) ) AND ( AFFILCOUNTRY ( canada ) ) AND ( LIMIT-TO ( PUBYEAR , 2019 ) OR LIMIT-TO ( PUBYEAR , 2018 ) OR LIMIT-TO ( PUBYEAR , 2017 ) OR LIMIT-TO ( PUBYEAR , 2016 ) OR LIMIT-TO ( PUBYEAR , 2015 ) OR LIMIT-TO ( PUBYEAR , 2014 ) OR LIMIT-TO ( PUBYEAR , 2013 ) OR LIMIT-TO ( PUBYEAR , 2012 ) OR LIMIT-TO ( PUBYEAR , 2011 ) OR LIMIT-TO ( PUBYEAR , 2010 ) ) AND ( EXCLUDE ( DOCTYPE , "ch" ) OR EXCLUDE ( DOCTYPE , "cp" ) ) AND ( LIMIT-TO ( PUBSTAGE , "final" ) ) AND ( LIMIT-TO ( LANGUAGE , "English" ) ) AND ( LIMIT-TO ( SRCTYPE , "j" ) )

**Web of Science**

TOPIC: ((“Chagas disease” OR “American trypanosomiasis” OR “Trypanosoma cruzi” OR “Triatominae” OR “kissing bugs” OR "T. cruzi Infection" OR "South American Trypanosomiasis" OR "Disease, Chagas" OR "Trypanosomiasis, American" OR "Trypanosomiasis, South American" OR "Chagas' Disease" OR "Trypanosoma cruzi"))

Refined by: PUBLICATION YEARS: ( 2019 OR 2018 OR 2017 OR 2016 OR 2015 OR 2014 OR 2013 OR 2012 OR 2011 OR 2010 ) AND [excluding] DOCUMENT TYPES: ( BOOK REVIEW OR MEETING ABSTRACT OR PROCEEDINGS PAPER OR BOOK ) AND COUNTRIES/REGIONS: ( CANADA ) AND LANGUAGES: ( ENGLISH )

Timespan: All years. Indexes: SCI-EXPANDED, SSCI, A&HCI, CPCI-S, CPCI-SSH, BKCI-S, BKCI-SSH, ESCI, CCR-EXPANDED, IC.

**8) Mycetoma, chromoblastomycosis and other deep mycoses**

**Date of Search: September 18th, 2020**

**Scopus**

TITLE-ABS-KEY ( ( "Chromoblastomycosis" OR "Fonsecaea pedrosoi" OR "Cladophialophora carrionii" OR "Phialophora verrucosa" OR "Fonsecaea compacta" OR "Mycetoma" OR "Mycetoma belt" OR "Deep mycoses" ) ) AND ( AFFILCOUNTRY ( canada ) ) AND ( LIMIT-TO ( PUBYEAR , 2018 ) OR LIMIT-TO ( PUBYEAR , 2017 ) OR LIMIT-TO ( PUBYEAR , 2015 ) OR LIMIT-TO ( PUBYEAR , 2014 ) OR LIMIT-TO ( PUBYEAR , 2013 ) OR LIMIT-TO ( PUBYEAR , 2012 ) OR LIMIT-TO ( PUBYEAR , 2010 ) ) AND ( LIMIT-TO ( PUBSTAGE , "final" ) ) AND ( LIMIT-TO ( DOCTYPE , "ar" ) OR LIMIT-TO ( DOCTYPE , "re" ) OR LIMIT-TO ( DOCTYPE , "sh" ) ) AND ( LIMIT-TO ( LANGUAGE , "English" ) ) AND ( LIMIT-TO ( SRCTYPE , "j" ) )

**Web of Science**

TOPIC: ((“Chromoblastomycosis” OR “Fonsecaea pedrosoi” OR “Cladophialophora carrionii” OR “Phialophora verrucosa” OR “Fonsecaea compacta” OR “Mycetoma” OR “Mycetoma belt” OR “Deep mycoses”))

Refined by: PUBLICATION YEARS: ( 2019 OR 2018 OR 2017 OR 2016 OR 2015 OR 2014 OR 2013 OR 2012 OR 2011 OR 2010 ) AND [excluding] DOCUMENT TYPES: ( BOOK CHAPTER OR MEETING ABSTRACT OR PROCEEDINGS PAPER ) AND COUNTRIES/REGIONS: ( CANADA ) AND LANGUAGES: ( ENGLISH )

Timespan: All years. Indexes: SCI-EXPANDED, SSCI, A&HCI, CPCI-S, CPCI-SSH, BKCI-S, BKCI-SSH, ESCI, CCR-EXPANDED, IC.

**9) Taeniasis/Cysticercosis**

**Date of Search: September 18th, 2020**

**Scopus**

TITLE-ABS-KEY ( ( "cysticercosis" OR "Taenia solium" OR "taeniasis" OR "pork tapeworm" OR "human cysticercosis" OR "neurocysticercosis" OR "T. solium" OR "Taeniasis/cysticercosis" ) ) AND ( AFFILCOUNTRY ( canada ) ) AND ( LIMIT-TO ( PUBYEAR , 2019 ) OR LIMIT-TO ( PUBYEAR , 2018 ) OR LIMIT-TO ( PUBYEAR , 2017 ) OR LIMIT-TO ( PUBYEAR , 2016 ) OR LIMIT-TO ( PUBYEAR , 2015 ) OR LIMIT-TO ( PUBYEAR , 2014 ) OR LIMIT-TO ( PUBYEAR , 2013 ) OR LIMIT-TO ( PUBYEAR , 2012 ) OR LIMIT-TO ( PUBYEAR , 2011 ) OR LIMIT-TO ( PUBYEAR , 2010 ) ) AND ( EXCLUDE ( DOCTYPE , "ch" ) ) AND ( LIMIT-TO ( PUBSTAGE , "final" ) ) AND ( LIMIT-TO ( LANGUAGE , "English" ) OR LIMIT-TO ( LANGUAGE , "French" ) ) AND ( LIMIT-TO ( SRCTYPE , "j" ) )

**Web of Science**

TOPIC: ((“cysticercosis” OR “Taenia solium” OR “taeniasis” OR “pork tapeworm”OR “human cysticercosis” OR “neurocysticercosis” OR “T. solium” OR “Taeniasis/cysticercosis”))

Refined by: PUBLICATION YEARS: ( 2019 OR 2018 OR 2017 OR 2016 OR 2015 OR 2014 OR 2013 OR 2012 OR 2011 OR 2010 ) AND [excluding] DOCUMENT TYPES: ( BOOK CHAPTER OR MEETING ABSTRACT OR PROCEEDINGS PAPER ) AND COUNTRIES/REGIONS: ( CANADA ) AND LANGUAGES: ( ENGLISH )

Timespan: All years. Indexes: SCI-EXPANDED, SSCI, A&HCI, CPCI-S, CPCI-SSH, BKCI-S, BKCI-SSH, ESCI, CCR-EXPANDED, IC.

**10) Dengue Fever and chikungunya**

**Date of Search: October 18th, 2020**

**Scopus**

TITLE-ABS-KEY ( "Dengue Fever" OR "Dengue" OR "Dengue virus" OR "breakbone fever" OR "dengue shock syndrome" OR "Chikungunya" OR "chikungunya virus" OR "CHIKV" ) AND ( AFFILCOUNTRY ( canada ) ) AND ( LIMIT-TO ( PUBYEAR , 2019 ) OR LIMIT-TO ( PUBYEAR , 2018 ) OR LIMIT-TO ( PUBYEAR , 2017 ) OR LIMIT-TO ( PUBYEAR , 2016 ) OR LIMIT-TO ( PUBYEAR , 2015 ) OR LIMIT-TO ( PUBYEAR , 2014 ) OR LIMIT-TO ( PUBYEAR , 2013 ) OR LIMIT-TO ( PUBYEAR , 2012 ) OR LIMIT-TO ( PUBYEAR , 2011 ) OR LIMIT-TO ( PUBYEAR , 2010 ) ) AND ( LIMIT-TO ( PUBSTAGE , "final" ) ) AND ( LIMIT-TO ( DOCTYPE , "ar" ) OR LIMIT-TO ( DOCTYPE , "re" ) OR LIMIT-TO ( DOCTYPE , "le" ) OR LIMIT-TO ( DOCTYPE , "no" ) OR LIMIT-TO ( DOCTYPE , "ed" ) OR LIMIT-TO ( DOCTYPE , "sh" ) OR LIMIT-TO ( DOCTYPE , "er" ) ) AND ( LIMIT-TO ( LANGUAGE , "English" ) OR LIMIT-TO ( LANGUAGE , "French" ) ) AND ( LIMIT-TO ( SRCTYPE , "j" ) )

**Web of Science**

TOPIC: ("Dengue Fever" OR "Dengue" OR "Dengue virus" OR "breakbone fever" OR "dengue shock syndrome" OR "Chikungunya" OR "chikungunya virus" OR "CHIKV")

Refined by: PUBLICATION YEARS: ( 2019 OR 2018 OR 2017 OR 2016 OR 2015 OR 2014 OR 2013 OR 2012 OR 2011 OR 2010 ) AND COUNTRIES/REGIONS: ( CANADA ) AND [excluding] DOCUMENT TYPES: ( PROCEEDINGS PAPER OR BOOK REVIEW OR BOOK CHAPTER OR MEETING ABSTRACT ) AND LANGUAGES: ( ENGLISH OR FRENCH )

Timespan: All years. Indexes: SCI-EXPANDED, SSCI, A&HCI, CPCI-S, CPCI-SSH, BKCI-S, BKCI-SSH, ESCI, CCR-EXPANDED, IC.

**11) Dracunculiasis (Guinea Worm Disease)**

**Date of Search: September 21st, 2020**

**Scopus**

TITLE-ABS-KEY ( "Dracunculiasis*" OR "Guinea Worm Disease" OR "Guinea Worm" OR "Dracunculus medinensis" OR "Guinea-worm" OR "Dracunculo*" OR "Dracontiasis" OR "Medina Worm" OR "Dracunculiasis*" OR "Dracunculo*" OR "Dracontiasis" OR "Medina Worm" OR "Dracunculiases" OR "Guinea Worm Infection" OR "Infection, Guinea Worm" ) AND ( AFFILCOUNTRY ( canada ) ) AND ( LIMIT-TO ( PUBYEAR , 2018 ) OR LIMIT-TO ( PUBYEAR , 2017 ) OR LIMIT-TO ( PUBYEAR , 2016 ) OR LIMIT-TO ( PUBYEAR , 2015 ) OR LIMIT-TO ( PUBYEAR , 2014 ) OR LIMIT-TO ( PUBYEAR , 2013 ) OR LIMIT-TO ( PUBYEAR , 2012 ) OR LIMIT-TO ( PUBYEAR , 2011 ) OR LIMIT-TO ( PUBYEAR , 2010 ) ) AND ( LIMIT-TO ( PUBSTAGE , "final" ) ) AND ( LIMIT-TO ( DOCTYPE , "ar" ) OR LIMIT-TO ( DOCTYPE , "re" ) OR LIMIT-TO ( DOCTYPE , "le" ) ) AND ( LIMIT-TO ( LANGUAGE , "English" ) ) AND ( LIMIT-TO ( SRCTYPE , "j" ) )

**Web of Science**

TOPIC: ("Dracunculiasis*" OR "Guinea Worm Disease" OR "Guinea Worm" OR "Dracunculus medinensis" OR "Guinea-worm" OR "Dracunculo*" OR "Dracontiasis" OR "Medina Worm" OR "Dracunculiasis*" OR "Dracunculo*" OR "Dracontiasis" OR "Medina Worm" OR "Dracunculiases" OR "Guinea Worm Infection" OR "Infection, Guinea Worm")

Refined by: PUBLICATION YEARS: ( 2019 OR 2018 OR 2017 OR 2016 OR 2015 OR 2014 OR 2013 OR 2012 OR 2011 OR 2010 ) AND [excluding] DOCUMENT TYPES: ( MEETING ABSTRACT OR BOOK CHAPTER OR PROCEEDINGS PAPER OR BOOK ) AND COUNTRIES/REGIONS: ( CANADA ) AND LANGUAGES: ( ENGLISH )

Timespan: All years. Indexes: SCI-EXPANDED, SSCI, A&HCI, CPCI-S, CPCI-SSH, BKCI-S, BKCI-SSH, ESCI, CCR-EXPANDED, IC.

**12) Snakebite envenoming**

**Date of Search: September 21st, 2020**

**Scopus**

TITLE-ABS-KEY ( "Snakebite envenoming" OR "snakebite Disease" OR "venomous snake" OR "Snake bite" ) AND ( AFFILCOUNTRY ( canada ) ) AND ( LIMIT-TO ( PUBYEAR , 2019 ) OR LIMIT-TO ( PUBYEAR , 2018 ) OR LIMIT-TO ( PUBYEAR , 2017 ) OR LIMIT-TO ( PUBYEAR , 2015 ) OR LIMIT-TO ( PUBYEAR , 2014 ) OR LIMIT-TO ( PUBYEAR , 2013 ) OR LIMIT-TO ( PUBYEAR , 2012 ) OR LIMIT-TO ( PUBYEAR , 2011 ) OR LIMIT-TO ( PUBYEAR , 2010 ) ) AND ( LIMIT-TO ( PUBSTAGE , "final" ) ) AND ( LIMIT-TO ( DOCTYPE , "ar" ) OR LIMIT-TO ( DOCTYPE , "no" ) OR LIMIT-TO ( DOCTYPE , "re" ) ) AND ( LIMIT-TO ( LANGUAGE , "English" ) ) AND ( LIMIT-TO ( SRCTYPE , "j" ) )

**Web of Science**

TOPIC: ("Snakebite envenoming" OR "snakebite Disease" OR "venomous snake" OR "Snake bite")

Refined by: PUBLICATION YEARS: ( 2019 OR 2018 OR 2017 OR 2016 OR 2015 OR 2014 OR 2013 OR 2012 OR 2011 OR 2010 ) AND [excluding] DOCUMENT TYPES: ( MEETING ABSTRACT OR PROCEEDINGS PAPER OR BOOK CHAPTER OR BOOK REVIEW OR POETRY ) AND COUNTRIES/REGIONS: ( CANADA ) AND LANGUAGES: ( ENGLISH )

Timespan: All years. Indexes: SCI-EXPANDED, SSCI, A&HCI, CPCI-S, CPCI-SSH, BKCI-S, BKCI-SSH, ESCI, CCR-EXPANDED, IC.

**13) Human African Trypanosomiasis (African Sleeping Sickness)**

**Date of Search: September 21st, 2020**

**Scopus**

TITLE-ABS-KEY ( "Human African Trypanosomiasis" OR "African Sleeping Sickness" OR "sleeping sickness" OR "Trypanosoma brucei" OR "Trypanosoma brucei gambiense" OR "Trypanosoma brucei rhodesiense" OR "African trypanosomiasis*" OR "Trypanosomiasis, African" OR "African Trypanosomiases" OR "Trypanosomiases, African" OR "Sleeping Sickness, African" OR "Nagana" OR "Trypanosomes" OR "T. gambiense" OR "T. rhodesiense" ) AND ( AFFILCOUNTRY ( canada ) ) AND ( LIMIT-TO ( PUBYEAR , 2019 ) OR LIMIT-TO ( PUBYEAR , 2018 ) OR LIMIT-TO ( PUBYEAR , 2017 ) OR LIMIT-TO ( PUBYEAR , 2016 ) OR LIMIT-TO ( PUBYEAR , 2015 ) OR LIMIT-TO ( PUBYEAR , 2014 ) OR LIMIT-TO ( PUBYEAR , 2013 ) OR LIMIT-TO ( PUBYEAR , 2012 ) OR LIMIT-TO ( PUBYEAR , 2011 ) OR LIMIT-TO ( PUBYEAR , 2010 ) ) AND ( EXCLUDE ( DOCTYPE , "ch" ) OR EXCLUDE ( DOCTYPE , "cp" ) ) AND ( LIMIT-TO ( PUBSTAGE , "final" ) ) AND ( LIMIT-TO ( LANGUAGE , "English" ) ) AND ( LIMIT-TO ( SRCTYPE , "j" ) )

**Web of Science**

TOPIC: ("Human African Trypanosomiasis" OR "African Sleeping Sickness" OR "sleeping sickness" OR "Trypanosoma brucei" OR "Trypanosoma brucei gambiense" OR "Trypanosoma brucei rhodesiense" OR "African trypanosomiasis*" OR "Trypanosomiasis, African" OR "African Trypanosomiases" OR "Trypanosomiases, African" OR "Sleeping Sickness, African" OR "Nagana" OR "Trypanosomes" OR "T. gambiense" OR "T. rhodesiense")

Refined by: PUBLICATION YEARS: ( 2019 OR 2018 OR 2017 OR 2016 OR 2015 OR 2014 OR 2013 OR 2012 OR 2011 OR 2010 ) AND [excluding] DOCUMENT TYPES: ( PROCEEDINGS PAPER OR BOOK REVIEW OR MEETING ABSTRACT OR BOOK CHAPTER OR BOOK ) AND COUNTRIES/REGIONS: ( CANADA ) AND LANGUAGES: ( ENGLISH )

Timespan: All years. Indexes: SCI-EXPANDED, SSCI, A&HCI, CPCI-S, CPCI-SSH, BKCI-S, BKCI-SSH, ESCI, CCR-EXPANDED, IC.

**14) Leishmaniasis**

**Date of Search: September 21st, 2020**

**Scopus**

TITLE-ABS-KEY ( "Leishmaniasis" OR "Leishmania parasites" OR "mucocutaneous leishmaniasis" OR "Leishmania Infection" OR "Black Fever" OR "Oriental sore" OR "Delhi boil" OR "Visceral leishmaniasis" OR "cutaneous leishmaniasis" OR "mucocutaneouso" OR "Leishmaniasis, Visceral" OR "Kala-Azar" OR "Kala Azar" OR "cutaneous leishmaniases" OR "leishmaniases, cutaneous" OR "sore, oriental" OR "leishmaniasis, old world" OR "old world leishmaniasis" OR "leishmaniasis, new world" OR "new world leishmaniasis" OR "leishmaniasis, american" OR "american leishmaniasis" OR "L.donovani" OR "L.infantum" OR "L.chagasi" OR "L.mexicana" OR "L.amazonensis" OR "L.venezuelensis" OR "L.tropica" OR "L.major" OR "L.aethiopica" OR "L.V. braziliensis" OR "L. V. guyanensis" OR "L. V. panamensis" OR "L.V. peruviana" ) AND ( AFFILCOUNTRY ( canada ) ) AND ( LIMIT-TO ( PUBYEAR , 2019 ) OR LIMIT-TO ( PUBYEAR , 2018 ) OR LIMIT-TO ( PUBYEAR , 2017 ) OR LIMIT-TO ( PUBYEAR , 2016 ) OR LIMIT-TO ( PUBYEAR , 2015 ) OR LIMIT-TO ( PUBYEAR , 2014 ) OR LIMIT-TO ( PUBYEAR , 2013 ) OR LIMIT-TO ( PUBYEAR , 2012 ) OR LIMIT-TO ( PUBYEAR , 2011 ) OR LIMIT-TO ( PUBYEAR , 2010 ) ) AND ( EXCLUDE ( DOCTYPE , "ch" ) OR EXCLUDE ( DOCTYPE , "cp" ) ) AND ( LIMIT-TO ( PUBSTAGE , "final" ) ) AND ( LIMIT-TO ( LANGUAGE , "English" ) OR LIMIT-TO ( LANGUAGE , "French" ) ) AND ( LIMIT-TO ( SRCTYPE , "j" ) )

**Web of Science**

TOPIC: ("Leishmaniasis" OR "Leishmania parasites" OR "mucocutaneous leishmaniasis" OR "Leishmania Infection" OR "Black Fever" OR "Oriental sore" OR "Delhi boil" OR "Visceral leishmaniasis" OR "cutaneous leishmaniasis" OR "mucocutaneouso" OR "Leishmaniasis, Visceral" OR "Kala-Azar" OR "Kala Azar" OR "cutaneous leishmaniases" OR "leishmaniases, cutaneous" OR "sore, oriental" OR "leishmaniasis, old world" OR "old world leishmaniasis" OR "leishmaniasis, new world" OR "new world leishmaniasis" OR "leishmaniasis, american" OR "american leishmaniasis" OR "L.donovani" OR "L.infantum" OR "L.chagasi" OR "L.mexicana" OR "L.amazonensis" OR "L.venezuelensis" OR "L.tropica" OR "L.major" OR "L.aethiopica" OR "L.V. braziliensis" OR "L. V. guyanensis" OR "L. V. panamensis" OR "L.V. peruviana")

Refined by: PUBLICATION YEARS: ( 2019 OR 2018 OR 2017 OR 2016 OR 2015 OR 2014 OR 2013 OR 2012 OR 2011 OR 2010 ) AND [excluding] DOCUMENT TYPES: ( BOOK CHAPTER OR BOOK REVIEW OR MEETING ABSTRACT OR PROCEEDINGS PAPER OR BOOK ) AND COUNTRIES/REGIONS: ( CANADA ) AND LANGUAGES: ( ENGLISH )

Timespan: All years. Indexes: SCI-EXPANDED, SSCI, A&HCI, CPCI-S, CPCI-SSH, BKCI-S, BKCI-SSH, ESCI, CCR-EXPANDED, IC.

**15) Foodborne trematodiases**

**Date of Search: September 21st, 2020**

**Scopus**

TITLE-ABS-KEY ( "Foodborne trematodiases" OR "Clonorchis" OR "Opisthorchis" OR "Fasciola" OR "Paragonimus" OR "trematodes" OR "Clonorchis sinensis" OR " Opisthorchis viverrini" OR "fishborne trematodes" OR "Paragonimus westermani" OR "Paragonimus heterotremus" OR "Paragonimus philippinensis" OR "Fasciola hepatica" OR "Fasciola gigantica" ) AND ( AFFILCOUNTRY ( canada ) ) AND ( LIMIT-TO ( PUBYEAR , 2019 ) OR LIMIT-TO ( PUBYEAR , 2018 ) OR LIMIT-TO ( PUBYEAR , 2017 ) OR LIMIT-TO ( PUBYEAR , 2016 ) OR LIMIT-TO ( PUBYEAR , 2015 ) OR LIMIT-TO ( PUBYEAR , 2014 ) OR LIMIT-TO ( PUBYEAR , 2013 ) OR LIMIT-TO ( PUBYEAR , 2012 ) OR LIMIT-TO ( PUBYEAR , 2011 ) OR LIMIT-TO ( PUBYEAR , 2010 ) ) AND ( EXCLUDE ( DOCTYPE , "ch" ) OR EXCLUDE ( DOCTYPE , "cp" ) ) AND ( LIMIT-TO ( PUBSTAGE , "final" ) ) AND ( LIMIT-TO ( LANGUAGE , "English" ) ) AND ( LIMIT-TO ( SRCTYPE , "j" ) )

**Web of Science**

TOPIC: ("Foodborne trematodiases" OR "Clonorchis" OR "Opisthorchis" OR "Fasciola" OR "Paragonimus" OR "trematodes" OR "Clonorchis sinensis" OR " Opisthorchis viverrini" OR "fishborne trematodes" OR "Paragonimus westermani" OR "Paragonimus heterotremus" OR "Paragonimus philippinensis" OR "Fasciola hepatica" OR "Fasciola gigantica")

Refined by: PUBLICATION YEARS: ( 2019 OR 2018 OR 2017 OR 2016 OR 2015 OR 2014 OR 2013 OR 2012 OR 2011 OR 2010 ) AND [excluding] DOCUMENT TYPES: ( PROCEEDINGS PAPER OR BOOK OR BOOK REVIEW OR MEETING ABSTRACT OR BOOK CHAPTER ) AND COUNTRIES/REGIONS: ( CANADA ) AND LANGUAGES: ( ENGLISH )

Timespan: All years. Indexes: SCI-EXPANDED, SSCI, A&HCI, CPCI-S, CPCI-SSH, BKCI-S, BKCI-SSH, ESCI, CCR-EXPANDED, IC.

**16) Leprosy (Hansen's disease)**

**Date of Search: September 22nd, 2020**

**Scopus**

TITLE-ABS-KEY ( "Leprosy" OR "Hansen's disease" OR "Mycobacterium leprae" OR "Leprosy Tuberculoid" OR "Leprosy, Neural" OR "Leprosy, Lepromatous" OR "Leprosy, Cutaneous" OR "Leprosy Modular" OR "Leprosy, Borderline" OR "Leprosy Dimorphous" OR "Leprosy Histoid" ) AND ( AFFILCOUNTRY ( canada ) ) AND ( LIMIT-TO ( PUBYEAR , 2019 ) OR LIMIT-TO ( PUBYEAR , 2018 ) OR LIMIT-TO ( PUBYEAR , 2017 ) OR LIMIT-TO ( PUBYEAR , 2016 ) OR LIMIT-TO ( PUBYEAR , 2015 ) OR LIMIT-TO ( PUBYEAR , 2014 ) OR LIMIT-TO ( PUBYEAR , 2013 ) OR LIMIT-TO ( PUBYEAR , 2012 ) OR LIMIT-TO ( PUBYEAR , 2011 ) OR LIMIT-TO ( PUBYEAR , 2010 ) ) AND ( EXCLUDE ( DOCTYPE , "ch" ) OR EXCLUDE ( DOCTYPE , "cp" ) ) AND ( LIMIT-TO ( PUBSTAGE , "final" ) ) AND ( LIMIT-TO ( LANGUAGE , "English" ) OR LIMIT-TO ( LANGUAGE , "French" ) ) AND ( LIMIT-TO ( SRCTYPE , "j" ) )

**Web of Science**

TOPIC: (“Leprosy” OR “Hansen's disease” OR “Mycobacterium leprae” OR "Leprosy Tuberculoid" OR "Leprosy, Neural" or "Leprosy, Lepromatous" OR "Leprosy, Cutaneous" OR "Leprosy Modular" or "Leprosy, Borderline" or "Leprosy Dimorphous" or "Leprosy Histoid")

Refined by: PUBLICATION YEARS: ( 2019 OR 2018 OR 2017 OR 2016 OR 2015 OR 2014 OR 2013 OR 2012 OR 2011 OR 2010 ) AND [excluding] DOCUMENT TYPES: ( BOOK CHAPTER OR RETRACTION OR PROCEEDINGS PAPER OR MEETING ABSTRACT OR BOOK REVIEW OR BOOK OR RETRACTED PUBLICATION ) AND LANGUAGES: ( ENGLISH OR FRENCH ) AND COUNTRIES/REGIONS: ( CANADA )

Timespan: All years. Indexes: SCI-EXPANDED, SSCI, A&HCI, CPCI-S, CPCI-SSH, BKCI-S, BKCI-SSH, ESCI, CCR-EXPANDED, IC.

**17) Rabies**

**Date of Search: September 22nd, 2020**

**Scopus**

TITLE-ABS-KEY ( "Rabies" OR "Furious rabies" OR "Paralytic rabies" OR "rabies virus" OR "Dog-mediated human rabies" OR "Dog-mediated rabies" ) AND ( AFFILCOUNTRY ( canada ) ) AND ( LIMIT-TO ( PUBYEAR , 2019 ) OR LIMIT-TO ( PUBYEAR , 2018 ) OR LIMIT-TO ( PUBYEAR , 2017 ) OR LIMIT-TO ( PUBYEAR , 2016 ) OR LIMIT-TO ( PUBYEAR , 2015 ) OR LIMIT-TO ( PUBYEAR , 2014 ) OR LIMIT-TO ( PUBYEAR , 2013 ) OR LIMIT-TO ( PUBYEAR , 2012 ) OR LIMIT-TO ( PUBYEAR , 2011 ) OR LIMIT-TO ( PUBYEAR , 2010 ) ) AND ( EXCLUDE ( DOCTYPE , "ch" ) OR EXCLUDE ( DOCTYPE , "bk" ) OR EXCLUDE ( DOCTYPE , "cp" ) ) AND ( LIMIT-TO ( PUBSTAGE , "final" ) ) AND ( LIMIT-TO ( LANGUAGE , "English" ) OR LIMIT-TO ( LANGUAGE , "French" ) ) AND ( LIMIT-TO ( SRCTYPE , "j" ) )

**Web of Science**

TOPIC: ("Rabies" OR "Furious rabies" OR "Paralytic rabies" OR "rabies virus" OR "Dog-mediated human rabies" OR "Dog-mediated rabies")

Refined by: PUBLICATION YEARS: ( 2019 OR 2018 OR 2017 OR 2016 OR 2015 OR 2014 OR 2013 OR 2012 OR 2011 OR 2010 ) AND [excluding] DOCUMENT TYPES: ( BOOK REVIEW OR BOOK OR MEETING ABSTRACT OR BOOK CHAPTER ) AND COUNTRIES/REGIONS: ( CANADA ) AND LANGUAGES: ( ENGLISH )

Timespan: All years. Indexes: SCI-EXPANDED, SSCI, A&HCI, CPCI-S, CPCI-SSH, BKCI-S, BKCI-SSH, ESCI, CCR-EXPANDED, IC.

**18) Scabies and other ectoparasites**

**Date of Search: September 22nd, 2020**

**Scopus**

TITLE-ABS-KEY ( "Scabies" OR "Sarcoptes scabiei var hominis" OR "human itch mite" OR "Crusted scabies" OR "Norwegian scabies" OR "Sarcoptes scabiei" OR "Human scabies" OR "Tungiasis" OR "Tunga penetrans" OR "T. trimamillata" OR "pulga de areia" OR "nigua" OR "pique" OR "bicho do pé" OR "bichodo porco" OR "jatecuba" OR "jigger" OR "sand flea" OR "chigoe" ) AND ( AFFILCOUNTRY ( canada ) ) AND ( LIMIT-TO ( PUBYEAR , 2019 ) OR LIMIT-TO ( PUBYEAR , 2018 ) OR LIMIT-TO ( PUBYEAR , 2017 ) OR LIMIT-TO ( PUBYEAR , 2016 ) OR LIMIT-TO ( PUBYEAR , 2015 ) OR LIMIT-TO ( PUBYEAR , 2014 ) OR LIMIT-TO ( PUBYEAR , 2013 ) OR LIMIT-TO ( PUBYEAR , 2012 ) OR LIMIT-TO ( PUBYEAR , 2011 ) OR LIMIT-TO ( PUBYEAR , 2010 ) ) AND ( EXCLUDE ( DOCTYPE , "ch" ) ) AND ( LIMIT-TO ( PUBSTAGE , "final" ) ) AND ( LIMIT-TO ( LANGUAGE , "English" ) OR LIMIT-TO ( LANGUAGE , "French" ) ) AND ( LIMIT-TO ( SRCTYPE , "j" ) )

**Web of Science**

TOPIC: (“Scabies” OR “Sarcoptes scabiei var hominis” OR “human itch mite” OR “Crusted scabies” OR “Norwegian scabies” OR “Sarcoptes scabiei” OR “Human scabies” OR “Tungiasis” OR “Tunga penetrans” OR “T. trimamillata” OR “pulga de areia” OR “nigua” OR “pique” OR “bicho do pé” OR “bichodo porco” OR “jatecuba” OR “jigger” OR “sand flea” OR “chigoe”)

Refined by: PUBLICATION YEARS: ( 2019 OR 2018 OR 2017 OR 2016 OR 2015 OR 2014 OR 2013 OR 2012 OR 2011 OR 2010 ) AND [excluding] DOCUMENT TYPES: ( PROCEEDINGS PAPER OR BOOK REVIEW OR BOOK CHAPTER OR MEETING ABSTRACT ) AND COUNTRIES/REGIONS: ( CANADA ) AND LANGUAGES: ( ENGLISH )

Timespan: All years. Indexes: SCI-EXPANDED, SSCI, A&HCI, CPCI-S, CPCI-SSH, BKCI-S, BKCI-SSH, ESCI, CCR-EXPANDED, IC.

**19) Soil-transmitted helminthiases**

**Date of Search: September 22nd, 2020**

**Scopus**

TITLE-ABS-KEY ( "Soil-transmitted helminthiases" OR "Soil-transmitted helminth" OR "Ascaris lumbricoides" OR "Trichuris trichiura" OR "Necator americanus" OR "Ancylostoma duodenale" OR "Ascaris" OR "whipworm" OR "Hookworm" OR "Ascariasis*" OR "Parasitic Roundworm Disease" OR "A.lumbricoide*" OR "Trichocephalus trichiuris" OR "trichuriasis" OR "Human Whipworm" OR "Trichocephaliasis" OR "Trichocephaliases" OR "Whipworm Disease" OR "New World hookworm" OR "N. americanus" OR "Old World hookworm" OR "hookworm infection" ) AND ( AFFILCOUNTRY ( canada ) ) AND "Human*" AND ( LIMIT-TO ( PUBYEAR , 2019 ) OR LIMIT-TO ( PUBYEAR , 2018 ) OR LIMIT-TO ( PUBYEAR , 2017 ) OR LIMIT-TO ( PUBYEAR , 2016 ) OR LIMIT-TO ( PUBYEAR , 2015 ) OR LIMIT-TO ( PUBYEAR , 2014 ) OR LIMIT-TO ( PUBYEAR , 2013 ) OR LIMIT-TO ( PUBYEAR , 2012 ) OR LIMIT-TO ( PUBYEAR , 2011 ) OR LIMIT-TO ( PUBYEAR , 2010 ) AND ( LIMIT-TO ( PUBSTAGE , "final" ) ) AND ( LIMIT-TO ( DOCTYPE , "ar" ) OR LIMIT-TO ( DOCTYPE , "re" ) OR LIMIT-TO ( DOCTYPE , "le" ) OR LIMIT-TO ( DOCTYPE , "no" ) ) AND ( LIMIT-TO ( LANGUAGE , "English" ) OR LIMIT-TO ( LANGUAGE , "French" ) ) AND ( LIMIT-TO ( SRCTYPE , "j" ) )

**Web of Science**

TOPIC: ("Soil-transmitted helminthiases" OR "Soil-transmitted helminth" OR "Ascaris lumbricoides" OR "Trichuris trichiura" OR "Necator americanus" OR "Ancylostoma duodenale" OR "Ascaris" OR "whipworm" OR "Hookworm" OR "Ascariasis*" OR "Parasitic Roundworm Disease" OR "A. lumbricoide*" OR "Trichocephalus trichiuris" OR "trichuriasis" OR "Human Whipworm" OR "Trichocephaliasis" OR "Trichocephaliases" OR "Whipworm Disease" OR "New World hookworm" OR "N. americanus" OR "Old World hookworm" OR "hookworm infection" AND "Human*")

Refined by: PUBLICATION YEARS: ( 2019 OR 2018 OR 2017 OR 2016 OR 2015 OR 2014 OR 2013 OR 2012 OR 2011 OR 2010 ) AND [excluding] DOCUMENT TYPES: ( PROCEEDINGS PAPER OR BOOK OR MEETING ABSTRACT OR BOOK REVIEW OR BOOK CHAPTER ) AND COUNTRIES/REGIONS: ( CANADA ) AND LANGUAGES: ( ENGLISH )

Timespan: All years. Indexes: SCI-EXPANDED, SSCI, A&HCI, CPCI-S, CPCI-SSH, BKCI-S, BKCI-SSH, ESCI, CCR-EXPANDED, IC.

**20) Yaws (Endemic treponematoses)**

**Date of Search: September 22nd, 2020**

**Scopus**

TITLE-ABS-KEY ( "Yaws" OR "Endemic treponematoses" OR "Treponema pertenue" OR "Secondary yaws" OR "human yaws" OR "pertenue" ) AND ( AFFILCOUNTRY ( canada ) ) AND NOT ( "memory" OR "truck*" OR "yaw rate" OR "Drive*" OR "yaw angle*" OR "vehicle*" OR " yaw movement*" OR "Yaw plane*" OR "orientation yaw" OR "orientation" OR "head yaw" OR "laparoscopic surgery" OR "ship*" OR "brachytherapy" OR "Interfractional Errors" OR "Radiotherapy" OR "Wind" OR "Aerodynamics" OR "kinematics" OR "Paddlefish" OR "autopilot" OR "dynamics" OR "face recognition" OR "postural" OR "adhesives" OR "Backpack system" ) AND ( LIMIT-TO ( PUBYEAR , 2019 ) OR LIMIT-TO ( PUBYEAR , 2018 ) OR LIMIT-TO ( PUBYEAR , 2017 ) OR LIMIT-TO ( PUBYEAR , 2016 ) OR LIMIT-TO ( PUBYEAR , 2015 ) OR LIMIT-TO ( PUBYEAR , 2014 ) OR LIMIT-TO ( PUBYEAR , 2013 ) OR LIMIT-TO ( PUBYEAR , 2010 ) ) AND ( LIMIT-TO ( PUBSTAGE , "final" ) ) AND ( LIMIT-TO ( DOCTYPE , "ar" ) OR LIMIT-TO ( DOCTYPE , "le" ) OR LIMIT-TO ( DOCTYPE , "re" ) ) AND ( LIMIT-TO ( LANGUAGE , "English" ) ) AND ( LIMIT-TO ( SRCTYPE , "j" ) )

**Web of Science**

TOPIC: ("Yaws" OR "Endemic treponematoses" OR "Treponema pertenue" OR "Secondary yaws" OR "human yaws" OR "pertenue")

Refined by: PUBLICATION YEARS: ( 2019 OR 2018 OR 2017 OR 2016 OR 2015 OR 2014 OR 2013 OR 2012 OR 2011 OR 2010 ) AND [excluding] DOCUMENT TYPES: ( MEETING ABSTRACT OR BOOK REVIEW OR PROCEEDINGS PAPER OR BOOK CHAPTER ) AND COUNTRIES/REGIONS: ( CANADA ) AND LANGUAGES: ( ENGLISH )

Timespan: All years. Indexes: SCI-EXPANDED, SSCI, A&HCI, CPCI-S, CPCI-SSH, BKCI-S, BKCI-SSH, ESCI, CCR-EXPANDED, IC.

**21) Neglected Tropical Diseases**

**Date of search: October 26th, 2020**

**Scopus**

TITLE-ABS-KEY ( ( "Neglected Tropical Diseases" OR "NTD" OR "NTDs" OR "Neglected Tropical Disease" ) ) AND ( AFFILCOUNTRY ( canada ) ) AND ( LIMIT-TO ( PUBYEAR , 2019 ) OR LIMIT-TO ( PUBYEAR , 2018 ) OR LIMIT-TO ( PUBYEAR , 2017 ) OR LIMIT-TO ( PUBYEAR , 2016 ) OR LIMIT-TO ( PUBYEAR , 2015 ) OR LIMIT-TO ( PUBYEAR , 2014 ) OR LIMIT-TO ( PUBYEAR , 2013 ) OR LIMIT-TO ( PUBYEAR , 2012 ) OR LIMIT-TO ( PUBYEAR , 2011 ) OR LIMIT-TO ( PUBYEAR , 2010 ) ) AND ( LIMIT-TO ( PUBSTAGE , "final" ) ) AND ( LIMIT-TO ( DOCTYPE , "ar" ) OR LIMIT-TO ( DOCTYPE , "re" ) OR LIMIT-TO ( DOCTYPE , "no" ) OR LIMIT-TO ( DOCTYPE , "ed" ) OR LIMIT-TO ( DOCTYPE , "er" ) OR LIMIT-TO ( DOCTYPE , "sh" ) OR LIMIT-TO ( DOCTYPE , "Undefined" ) ) AND ( LIMIT-TO ( LANGUAGE , "English" ) ) AND ( LIMIT-TO ( SRCTYPE , "j" ) )

**Web of Science**

TOPIC: ("Neglected Tropical Diseases" OR "NTD" OR "NTDs" OR "Neglected Tropical Disease")

Refined by: PUBLICATION YEARS: ( 2019 OR 2018 OR 2017 OR 2016 OR 2015 OR 2014 OR 2013 OR 2012 OR 2011 OR 2010 ) AND [excluding] DOCUMENT TYPES: ( MEETING ABSTRACT OR BOOK OR BOOK CHAPTER OR BIOGRAPHICAL ITEM OR PROCEEDINGS PAPER OR BOOK REVIEW ) AND COUNTRIES/REGIONS: ( CANADA ) AND LANGUAGES: ( ENGLISH )

Timespan: All years. Indexes: SCI-EXPANDED, SSCI, A&HCI, CPCI-S, CPCI-SSH, BKCI-S, BKCI-SSH, ESCI, CCR-EXPANDED, IC.
